# Interprofessional Collaboration Between Community Health Workers and Pharmacists

**DOI:** 10.1111/hex.70538

**Published:** 2026-01-19

**Authors:** Carole Bandiera, Megan Darwood, Sabuj Kanti Mistry, Elizabeth Harris, Mark F. Harris, Parisa Aslani

**Affiliations:** ^1^ Faculty of Medicine and Health, Sydney Pharmacy School University of Sydney Sydney New South Wales Australia; ^2^ The University of Nottingham Nottingham UK; ^3^ School of Population Health University of New South Wales Sydney New South Wales Australia; ^4^ International Centre for Future Health Systems University of New South Wales Sydney New South Wales Australia

**Keywords:** collaborative practice, community health workers, health navigators, interprofessional collaboration, interprofessional collaboration challenges, interprofessional collaboration strategies, pharmacists

## Abstract

**Background:**

Community health workers (CHWs) can bridge the gap between health and social services and the communities they serve. CHWs can work collaboratively with healthcare professionals, such as pharmacists, in addressing peoples' social determinants of health. However, little is known about how CHWs and pharmacists collaborate. We aimed to explore the interprofessional collaboration between pharmacists and CHWs in Australia and New Zealand and identify the challenges and strategies in their collaborative practice.

**Methods:**

Semi‐structured interviews were conducted with pharmacists and CHWs in Australia and New Zealand, to explore (i) CHW‐pharmacist interprofessional collaboration experiences, (ii) how pharmacists perceived CHWs' roles and vice‐versa, (iii), challenges to collaborative practices and (iv) strategies to foster their collaboration. Interviews took around half‐an‐hour and were audio‐recorded, transcribed verbatim, and inductively thematically analysed.

**Results:**

Twenty‐nine participants (16 pharmacists and 13 CHWs) were interviewed, 19 worked in Australia and 10 in New Zealand. Participants reported that CHWs connected the patient and the pharmacist and helped bridge cultural gaps, shared patient‐related information with the pharmacist, supported patient medication management and adherence, and referred patients to pharmacists. CHWs helped patients to take up pharmacy services and reinforced pharmacists' recommendations. There was a lack of clarity regarding each other's role, but pharmacists and CHWs acknowledged the benefit of each other's roles. Key challenges to collaborative practices were poor communication between CHWs and pharmacists, and lack of guidelines on the collaborative practices. Strategies to promote collaboration included clarification of their roles, improving the working relationship and knowledge of the mutual benefits of collaboration.

**Conclusions:**

The study identified some collaborative practices between CHWs and pharmacists. However, challenges remain, including a lack of clarity regarding CHWs' roles by pharmacists. Future research should focus on improving knowledge of the mutual benefits of collaboration, and codesigning a CHW‐pharmacist collaborative intervention with guidelines to standardise and foster collaboration.

**Patient or Public Contribution:**

Patients, service users, care‐givers, people with lived experience or members of the public were not involved in the study design or conduct of study, analysis or interpretation of the data or in preparation of the manuscript.

**Abbreviations**
AUD: Australian dollars
CHWs: community health workers
COREQ: consolidated criteria for reporting qualitative research
DAAs: dse administration aids
GP: general practitioner
IQR: interquartile range
NZD: New Zealand dollars
Pharm.: pharmacists
RedCap: Research Electronic Data Capture
SD: standard deviation
SDoH: social determinants of health

## 1 Introduction

According to the World Health Organization, interprofessional collaboration occurs when two or more health professionals from different professional backgrounds work together and learn from each other to synergistically improve the quality of patient care and ultimately patient health outcomes [1]. Interprofessional collaboration has been recognised and recommended internationally for better healthcare systems, and interprofessional education has been integrated in students' curricula to prepare future health professionals to collaborate effectively as part of multidisciplinary teams [1, 2, 3]. Key strategies to promote interprofessional collaboration include role clarification, effective communication, team functioning, collaborative leadership, interprofessional conflict resolution and mutual respect, trust and shared decision making in order to deliver patient‐centred care [3]. The collaborative practice between different types of health professionals shows promising impacts on patient outcomes, but evaluating these effects has been challenging [4, 5, 6, 7].

Interprofessional collaboration between physicians and nurses has been commonly reported [8]. The interprofessional collaboration with community health workers (CHWs) has been reported mostly with physicians, nurses and dietitians [9]; the collaboration between CHWs and health professionals including social workers or pharmacists has been rarely studied [9, 10]. Little is known about the collaborative practices between pharmacists and CHWs [10, 11].

CHWs are health workers who bridge the gap between health and social services and the community they serve, mostly priority populations (e.g., people living in remote areas, marginalised people, minorities, individuals having limited access to services) [12, 13, 14]. CHWs have a variety of roles, ranging from facilitating patients' access to services, providing health education and behaviour change motivation, improving the relationship between patients and health professionals, and delivering diagnostic, treatment, clinical care and psychosocial support [13]. With cultural diversity growing in Australia and New Zealand [15, 16], and clinical professional staff shortages becoming critical, particularly in some remote regions [17, 18, 19], CHWs can become instrumental in supporting priority and underserved patients with their health and social care.

On the other hand, pharmacists are accessible health professionals, and their roles have been expanded from traditionally delivering medications to providing pharmaceutical care to optimise quality use of medicines [20, 21]. While pharmacists deliver medication reviews and support patient medication management and adherence, they may experience challenges in addressing patients' social determinants of health (SDoH) that can impact patients' medication use—such as low income, access to health services, unemployment—as well as integrating SDoH as part of their patient‐centred services [22, 23]. Therefore, it would be important to better understand the collaborative practices between CHWs and pharmacists, and how patients may be best supported and connected to community and social resources by pharmacists and CHWs working together [24].

There is potential for the CHW‐pharmacist collaboration to improve patient health outcomes and health equity [10, 11]. There are few reported studies that have evaluated the collaboration between pharmacists and CHWs in the United States of America [10, 11]. Interventions involving CHW‐pharmacist collaboration include medication reviews, medication therapy management, medication reconciliation, support for patient medication adherence, disease prevention and addressing SDoH [10, 11].

Some studies have reported either pharmacists' or CHWs' perceptions of their collaborative practices [25, 26, 27], and the challenges and facilitators of collaboration have been reported at different levels. Barriers to collaboration include maintaining patient confidentiality, lack of clarity of CHWs' roles, inconsistent communication between pharmacists and CHWs or work‐related scheduling conflicts [25, 28]. However, pharmacists have reported positive experiences and outcomes when working with CHWs, e.g., in understanding patients' way of life, facilitating communication between patients and clinicians, supporting patient medication adherence, improving patient empowerment and increasing healthcare utilisation [25, 26]. CHWs have reported that pharmacists are a trusted source of information [27].

Globally, there are few reported studies on interprofessional collaborative practice between CHWs and pharmacists, especially in Australia and New Zealand. Therefore, this study aimed to understand (i) the collaborative practices experienced by pharmacists and CHWs in Australia and New Zealand and (ii) the challenges encountered and potential strategies to improve their collaborative practice.

## 2 Methods

### 2.1 Ethical Considerations and Guidelines

The study was conducted in accordance with the declaration of Helsinki and was approved by the Human Research Ethics Committee of the University of Sydney (2024/HE000334) in June 2024. The standards for reporting qualitative research [29] were followed and the consolidated criteria for reporting qualitative research (COREQ) checklist was completed [30] (Supporting Information S1: Material S1).

### 2.2 Recruitment of Participants

The eligibility criteria were: (i) a CHW, the CHW supervisor or a pharmacist who has been working in health services (e.g., for pharmacists within a community pharmacy or a hospital; for CHWs within a community centre or a primary care organization) for at least 6 months in Australia or New Zealand and (ii) speaks English and does not require an interpreter.

Different approaches were used to recruit the participants from Australia and New‐Zealand: (i) potential participants were identified through the professional network of authors, (ii) the study flyer was displayed and advertised at the University and through the School of Pharmacy's newsletter, (iii) the study flyer was advertised through an Australian professional association's newsletter sent to pharmacists, (iv) some study participants promoted the study in their professional network, (v) the study was promoted on the professional platform LinkedIn (LinkedIn Corporation) and on closed pharmacy professional groups on Facebook.

The initial contact with potential participants to introduce the study was made over the phone, via email or video‐call by CB, along with sending the participant information statement. Written informed consent was obtained before data collection. Interviews were conducted either online using Zoom (Zoom Communications, Inc.) or Microsoft Teams videoconferencing, or in‐person, at an agreed time and in an appropriate place ensuring participant's privacy and confidentiality.

The recruitment stopped after data saturation was reached. Data saturation was defined as when no new codes or themes were generated by the researchers in at least two consecutive interviews, indicating that no further data collection was needed [31, 32]. According to a recent systematic review, data saturation in qualitative research is reached after 9–17 interviews [33].

### 2.3 Data Collection

Semi‐structured interviews were conducted by CB between July 2024 and February 2025.

Separate interview guides were developed individually for both CHWs and pharmacists (Supporting Information S2 and S3: Materials S2 and S3). Both interview guides were comprised of open‐ended questions exploring: (i) the professional background of participants, (ii) professional experiences when delivering care to vulnerable patients, (iii) how pharmacists perceived CHWs' roles and vice versa, (iv) lived experience of interprofessional collaboration with CHWs or pharmacists, (v) collaborative practice barriers, (vi) collaborative practice strategies, (vii) opinions about future collaborative practice and (viii) participants' opinions about a published collaborative CHW‐pharmacist practice model in addressing medication adherence [34]. This paper will focus on the themes identified from questions (i) to (vii). The results of point (viii) will be presented elsewhere.

Prior to data collection, both pharmacist and CHW interview guides were piloted with three academic pharmacists and three CHWs.

A short demographic survey (Supporting Information S4: Material S4) was completed by the participants before the start of the interview to collect information on gender, age, duration of work experience in health services and cultural background. All study forms (e.g., participant consent form, demographic data and receipt of the e‐voucher) were filled‐in by participants in hard copy or online, according to their preferences. Online consent was completed via Research Electronic Data Capture (RedCap, Vanderbilt University), a secure, web‐based software platform designed to support data capture for research studies [35].

The interviews were audio‐recorded with recorders or using Zoom (Zoom Communications, Inc.) or Microsoft Teams videoconferencing. CB collected field notes during the interviews. The transcription and coding of interviews were done alongside data collection. The interviews were transcribed verbatim and any identifying information was removed from the transcripts. All participants were offered to review their interview transcript, two pharmacists and seven CHWs reviewed their transcript and no significant changes or comments were made.

Participants were reimbursed for their time with an e‐voucher for a local grocery store in Australia or New Zealand (AUD 100 or NZD 120 for pharmacists and AUD 75 or NZD 90 for CHWs).

### 2.4 Data Analysis

The de‐identified transcripts were analysed through a reflexive thematic analysis and coded using the NVivo 14 software (QSR International Pty Ltd). We acknowledge the diversity in thematic analysis [36], and we followed Braun and Clarke's work to conduct a scientifically descriptive reflexive thematic analysis approach and an inductive process, acknowledging the researchers' subjectivity [37, 38]. We created the topic summaries by: (1) reading of raw data, i.e., transcribed verbatims, (2) iterative identification of text segments in line with the study aims and relevant content, (3) identification of text segments to create and develop codes and sub‐codes, (4) reduction of similar or redundant codes and sub‐codes, (5) iterative process of refining the codes as the study findings evolved, (6) creation of a coding tree that integrates important codes into categories and main themes and subthemes, and (7) interpretation of the theme development, acknowledging reflexivity and subjectivity.

The researcher's perspectives, beliefs, assumptions and experiences inevitably shaped the interpretation of the data and the analytic process. CB, who conducted the reflexive thematic analysis, is a pharmacist by training, which may have influenced the analytic process towards a better understanding of the needs, challenges and strategies for pharmacists within the CHW‐pharmacist collaboration. To ensure coding validity, enhance trustworthiness and limit potential data interpretation through a reflexive approach, the codes were discussed iteratively and reviewed with SKM (who is not a pharmacist by training), PA and MD. The codes were then reviewed by EH and MH who are also not pharmacists by training. They worked together on codes and categories identification, and discrepancies were discussed until consensus achieved. The results have been described narratively along with citations of relevant quotes.

## 3 Results

### 3.1 Included Participants

In total, 34 eligible participants were approached (18 pharmacists and 16 CHWs) and 29 participants (85%) agreed to participate, including 16/18 pharmacists (89%) and 13/16 CHWs (81%). Demographic data of participants are shown in Table 1.

**Table 1:** Participants' demographic data.

Demographic data (*n* = 29)	Pharmacists (*n* = 16)		CHWs (*n* = 13)	
Self‐reported professional position(s), *n* (%)				
	Hospital pharm.	5 (31)	Health navigator	4 (31)
	Academic pharm.	2 (13)	Community health navigator	2 (15)
	Community and academic pharm.	2 (13)	Cultural support worker	2 (15)
	Community and hospital pharm.	2 (13)	Bilingual community educator	2 (15)
	Community pharm.	2 (13)	Psychosocial support worker	1 (8)
	Community and research‐based pharm.	1 (6)	Supervisor health navigator	1 (8)
	Hospital and research‐based pharm.	1 (6)	Smokefree services coordinator	1 (8)
	Academic and hospital pharm.	1 (6)		
Self‐identified gender, *n* (%)				
Female	10 (62)		10 (77)	
Male	6 (38)		3 (23)	
Years of work experience in health, median, (IQR)				
	9 (6–19)		8 (3–17)	
Self‐identified cultural background in the checklist provided^a^, *n* (%)				
	Oceanian	2 (13)	Oceanian	2 (15)
	North‐West European	1 (6)	Other^b^, as defined by participants	11 (85)
	South‐East Asian	5 (31)		
	Other^c^, as defined by participants	8 (50)		
Age in years, median (IQR)				
	32 (30–43)		50 (40–59)	
Place of work, *n* (%)				
Australia	12 (75)		7 (54)	
New Zealand	4 (25)		6 (46)	

Abbreviations: IQR, interquartile range; pharm., pharmacist(s).

^a^ Some of the participants reported several cultural backgrounds.

^b^ Other include New Zealander, Bangladeshi, Chinese, Australian, Eastern Asian, Indian, Northern English, Maori, Irish, Samoa, Pacific Islander, Syrian.

^c^ Other include Australian, Aboriginal and Torres Strait Islander, Caucasian, New Zealander, Maori.

After the study was advertised on LinkedIn (LinkedIn Corporation), three interviews were conducted with fraudulent participants, falsely claiming to be pharmacists or CHWs [39]. The data collected from these three participants were not analysed.

Eleven interviews with pharmacists (69%) were online, three were face to face and two by phone. Five interviews with CHWs (38%) were online, six face to face and two by phone.

Data saturation was reached after 12/16 interviews with pharmacists and 10/13 interviews with CHWs. However, further interviews were conducted to confirm data saturation. Mean duration of the interviews was 31 min (standard deviation [SD] = 7) with pharmacists and 32 min (SD = 6) with CHWs.

### 3.2 Main Themes Identified

In total, 990 text segments were coded for 13 CHW interviews and 1583 text segments for 16 pharmacist interviews. The themes identified were (1) a range of collaborative activities, (2) perception of each other's roles, (3) challenges to collaboration, (4) strategies to facilitate, maintain or improve the collaboration.

**Figure 1:**
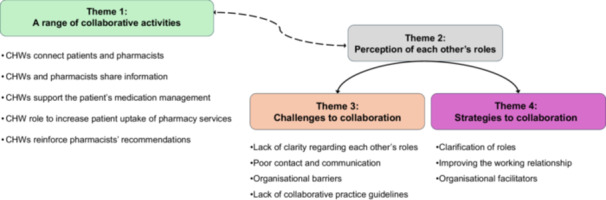
Main themes and subthemes representing the collaborative practices between CHWs and pharmacists together with the challenges and strategies, and the perception of each other's roles. *Note:* The dotted arrow indicates that the Themes 1 and 2 influence each other. The two undotted arrows show that the perception of each other's roles might be considered as barriers or strategies.

The main themes and their subthemes are summarised in a thematic map in Figure 1. The relevant quotes supported by the unique participant identifier, sometimes truncated for conciseness, are presented within the text and additional quotes are presented in Table 2. Of note, the quotes are all verbatim quotes and no grammatical corrections have been done.

**Table 2:** Pharmacists’ and community health workers’ additional quotes illustrating the subthemes.

Themes and subthemes	Pharmacists’ quotes	Community health workers’ quotes
Theme 1: A range of collaborative activities		
Frequency of the collaboration	1.1. *“I would probably say we don't have a lot of contact with the community health workers. (…) I probably feel like I haven't worked with them closely.”* P10	1.2. *“Uh, not directly [work with pharmacists]. I see some prescription it should be go to pharmacists but we didn't directly work with them.”* P09
CHWs connect patients and pharmacists	2.1. “*Usually for the first visit and the last visit, so doing the history and doing the discharge, I'll often enlist Aboriginal Health Worker because I also think they [the patients] feel more comfortable explaining and asking questions when they're around. So I think, once I have that relationship started with the Aboriginal Health Worker a lot of the time, even after that, when you go in, they'll* [the patients] *sort of link you back to having come in with Aboriginal Health workers. So, the relationship is starting to build. But again, it does depend on the patient, because some patients will react differently than other patients*.” P12	2.2. “*With Pacific patients, (…) once you able to link them in and just break the ice with a service, then they're more than happy to follow up themselves. Initially, it's always, the first kind of meeting with another service, they kind of get nervous about it, but once they rapport is built and they trust their service, they tend to just be like, “okay, I can do it myself”, then that's really rewarding in the health coaching role.”* P17
CHWs and pharmacists share information	*3.1. “I'm always slightly worried about confidentiality. When I'm reaching out to a carer about a patient without that patient's permission. I feel like the way that it's set up in Australia is that it's very clear I'm allowed to do that so long as I'm doing it as part of my duty of care. So, I read it, but if I've got a health reason to do that, then I absolutely can do that. But if I haven't got a good reason, I can't just pick up the phones of a carer and say, “Oh, they need another script to this”, unless I've already had standing permission from the patient to do that. So, that's one of the things that limits that collaboration… so in some cases it's no barrier at all, and in other cases it is.”* P14 3.2. “*Sometimes we have a little bit of backstory about patients (…). Mainly things about medications and where they might get them from, just other general things about them, about their medical history*.” P07	3.3. “*We have to share information sometimes… it could be with the medical team or the pharmacist, if we think it's something of a personal nature, no, we wouldn't, but if it's relating to their* [the patients] *care, yeah, we can share information. We understand privacy, of course. But you know, the clients understand (…) but if they were very private and they didn't want it* [that information is shared with pharmacists], *well, that would be different. So we respect their privacy, but at the same time, they understand we need to share some information*.” P25
CHWs support the patient's medication management	4.1 “We've got to work phone (…). I suppose they sort of refer to us sort of as much as we sort of refer to them. So sometimes we might call them first and say, “Would you mind coming in talking to this patient with us?” Or conversely, they might sort of say, “oh, this person doesn't really know much about their medications or wants to know a bit more about what's going on”, demand coming in. So mainly by phone.” P07	4.2. “*My clients, most of them discharged from the hospital. (…) In order to update the medication list, so I usually call the pharmacist (…). They always are over the phone, answer the questions. (…) If this client have a lot of issues with the medication, I usually trying to talk to the pharmacy.”* P03
CHW role to increase patient uptake of pharmacy services	*5.1. “So maybe it might be that we explain to the navigator how our services work, so they're, in turn, able to explain that to clients, or maybe explain why something can't be done a particular way, and the navigators may or may not, sort of translate that for the client, if it's helpful.” P30*	*5.2. “Sometimes I take the clients visit pharmacies together. And then sometimes I collaborate between the pharmacist, the GP, clients and myself together. (…) If it's like, suggest the clients to do the home medication review, that will (…) take longer. (…) I actually make the client aware there is a service* [the home medication review] *down there.” P03*
CHWs reinforce pharmacists’ recommendations	*6.1. “In a pharmacy setting, usually, if there's a carer or, like, support worker, (…) I would just check if the patient can understand a conversation with me, if not, if they're relying on the support worker entirely, I will just tell the support worker what to do, and, they will then relay the message onto the patient, or write it down for them, or help them remember it, set alarm* [to take medications], *or something like that.” P15*	*6.2. “Even if the pharmacist says, “Do this, do that”, but still people ask questions, especially those who are living in a different country and migrated here after a few years. Because sometimes people become habitual… for 20 years if they do the same thing. And then suddenly drastic change. So even they don't want to make mistake, but it just accidentally happens. So when the navigators are there, they just remind or, just follow up, that just keeps them, like refreshing the thought, “oh, yeah, I need to do that, oh, yes. I need to see my medication. Oh, ah, yes, I get to me, I need to get my review done with the pharmacist”. So that helps.” P04*
Theme 2: Perception of each other's roles		
	7.1. “*I think there's a real gap because I see a health navigator, I don't really know what they do, and I think that I'm not alone in that. So I think more education for health professionals of how we can better utilize a Health Navigator. Because sure, you know, like, they must be more than just transport. So I think it would be great to include them somehow*.” P22 7.2. “*The most important task that they do… I think it is bridging the gap, in helping the healthcare providers getting to the patients, in establishing, understanding, establishing rapport, establishing or any kind of misconception or miscommunication that can be debunked, they can also connect the patients to the required health systems, if that can also include the government system, for example, pension or concession that is required. There can be additional other health services that they can also because they are that center point so that they can connect the people to it as well.”* P13	*7.3. “Before I joined the New South Wales Health, (…) my understanding is, they [pharmacists] are dispensing the prescription medications to the client, to the public. And over the counter. So my understanding after the working as a CHWs, in the New South Wales Health, I actually found that pharmacist can provide much more services than then our thoughts). Yeah, so that actually can do a lot of helping clients understanding the kind of medicines they take. And also, the most important rules I learned from my work is… they can do the whole medication review. That's something most people I can say 99% of my clients never know this role before.”* P03 7.4. “*They process scripts and they get medication ready for patients, and they make up blister packs. They support people who go in asking questions about their health. People can go in there with a concern, and a pharmacist will come out and have a chat to them and suggest something or say maybe “you need to make a GP appointment”. So I can think… your role is pretty big*.” P22 *7.5.“I do realize that important research this is for the pharmacist. So I can't really say enough good things about the ones* [the pharmacists] *that we have. We are blessed. They are great.”* P22
Theme 3: Challenges to collaboration		
Lack of clarity regarding each other's roles	8.1. “*At least from my understanding, and this might show, demonstrate how naive I am (…), but what their* [CHWs] *scope of practice is because different community healthcare workers will provide different services, and so there's no maybe standardization of that. It might be difficult for clinicians to comprehend what is within scope and what's not*.” P18 *8.2. “And the other barriers could be maybe language barrier or the way they* [CHWs] *express things. If they don't have enough training, they might not know what they're exactly looking for and what to report back, you have to give them very specific instructions on…, or give or ask them very specific questions. And they may not also get the information they want from the patients, because they are not trained to do that.”* P15 8.3. “*I think there are very passive ones* [CHWs], *ones that just kind of, you know, stand there and listen and organize transport and don't do a lot else. And so they're not really actively participating in the management of that patient's health with me, I think, at least when I'm sort of saying things.”* P14 8.4. *“So that's kind of at one end of the spectrum, but there are some* [CHWs] *that really barely communicate with us at all, and then, then at the other end of the spectrum, it's the opposite, that they're giving us a lot of the information that we would need to be able to adjust our care for the patient. So some, I mean, I don't want to say disinterested, but some, I think, would think of themselves as, “look, look, I'm just the driver. Don't talk to me. I don't need to listen. I don't really care”. All the way to the other end where they kind of, they're the person that's keeping the patient out of hospital, really, the only reason that the patient's still here is because they're receiving that kind of care from a health worker but most of the time it lands somewhere between those two extremes. (…) It gets a bit clunky, particularly when you get differing levels of motivation (…) that's true for pharmacists as well.”* P14	8.5. “*I guess, because I'm not entirely sure how, or a little pharmacist here can do but if, if there was certain…, all vaccinations or certain types of medications that people could go to the pharmacist to talk about, instead of having to go the doctor, it would just relieve some of that pressure off our like, medical centers.”* P23 8.6. *“I do understand from my past experiences, (…) where they're* [CHWs] *speaking on behalf of the patient, other clinicians, let's say, like doctors and stuff, they may feel a bit of, there might be a bit of resistance, because I know as a health coach, there was a bit of resistance with the doctors I worked with, but then eventually they kind of understood my role. So I think it's just introducing to these clinicians and staff, the purpose of the model and what we're looking to establish and improve patient outcomes.”* P17
Poor contact and communication	9.1. “*So I do think, in that regards to Aboriginal Health Workers, I think they could do a better job of making themselves known, but I think pharmacists need to do a better job, all healthcare professionals need to do a better job of reaching out to try and find these people, because it's an untapped service and like people who do use these services really like them and like they actually find it helpful. So I think pharmacists need a better job of actually going out and finding these people.”* P32	*9.2. “And of course, we deal with the hospital pharmacy as well. So one of our greatest bugbears is that their department working very hard doesn't quite align itself with the person who's discharging from the hospital. So we find that very frustrating, because we help them out by taking patients home from hospital, and then we get there, only to find out that the pharmacist hasn't seen or is still working on the medication, and it's always late in the day (…). And so, it's just a processing issue, but we feel like we're a bugbear to the pharmacist down at the hospital”. P28*
Organisational barriers	10.1. “The only interaction we really have is when they [CHWs] would come into the pharmacy to pick up things for people. But I wouldn't say it's a hugely collaborative environment.” P24 *10.2. “I suppose it can be tricky when the patient isn't really on board sometimes. And so you know that, like setting up these systems with the carers to kind of supervise over is in the best interest of the patient, but the patient might think that they can still just manage their medicines from the boxes or whatever, even though they clearly can't.”* P10	*Refer to the quotes in the text*
Lack of collaborative practice guidelines	*11.1 “Isn't like a hospital setting like a, like a referral type system between each of us which we kind of do a moment with Aboriginal Health Workers, but it's not sort of documented anywhere, it's just us calling on the phone.” P07*	11.2 “*Normally I just refer to pharmacies because (…) I don't get paid for orders, liaising and all those things, you know, so I don't get involved unless until, like, you know, if they really want somebody from language specific or something, and if I know some pharmacists, then maybe I can just link that with that person. So that I just I just send a message to that pharmacists. And I might say, like, you know, there's somebody will turn up, they might need a language support, but I don't get in beyond point unless until it's required.” P04*
Theme 4: Strategies to collaboration		
Clarification of roles	*12.1. “The more we would know about each other's roles and scope and barriers, sorry and boundaries, the better (…). It's about learning about each other's skills and learning the scopes of practice of each provider. But also, I think pharmacists do need to appreciate the unique skills that navigators have and understand almost a case of walking in their shoes to understand what it's like to do their job successfully. So, what does good look like from a navigator perspective, what is it? And from a navigator perspective, what does a good pharmacist? (…) What makes a pharmacist good? (…) So a measure of reflection from both sides and adjustment of perspective.”* P30 12.2. “I *think obviously IPL, interprofessional learnings would be good. So actually having case scenarios with either doctors, nurses and community health workers and getting their role, because they already do it with doctors and nurses, and there's already case studies around that, so I don't think why we can't, again, do that with healthcare workers, and getting them involved and having their perspectives, and obviously, as I mentioned before, having actual practice in community healthcare workers coming in to the classroom and explaining their role and what they do, and again, maybe even having like workshops on it, about when you would seek out a worker or not, or you can kind of solve a problem by yourself.”* P32 12.3. “*Isn't like a hospital setting like a, like a referral type system between each of us which we kind of do a moment with Aboriginal Health Workers, but it's not sort of documented anywhere, it's just us calling on the phone. So I don't know maybe having some sort of mandatory referral process or… strategy…. I think there's just a bit of a misunderstanding about sort of the lack of what's actually out there*.” P07	*12.4. “We understand the pharmacists, like a lot of medical professionals, are very busy people, but we always like to let them know what we do. I think a lot of them are quite surprised when they find out as well.”* P25 12.5 “*Both of them need to (…) learn more about this role. Educate them about role, because it is so important role. But if the pharmacist don't know, they will be not interested. And as will of the community health workers don't know their role and (…) how they can help in the pharmacies, it will like not, not work. Educate both of them, and that, I think, if they trained, will have some programs to involve them in this role, it will be like (…) great.”. P09*
Improving the working relationship	13.1. “*What could help facilitate it… I guess, like an introduction as to who they are and a contact number to get directly through to them. Because sometimes, if you ring the* [CHW organization] *and you have to be put through to someone else, and someone else and someone else, probably direct contact line.”* P24	13.2. “*It is very informal, like we just bring them up, go and talk to them and say, “oh, what should we do? How can we help?” And the relationship's been great. (…)* *So I would say to the new navigator, organize a time to meet the pharmacist, let them know what you do, how to contact you, establish that relationship first, so that you can deal with them on a one on one basis.”*, P22
Organisational facilitators	14.1. “*They* [CHWs] *probably know the patient better than us, the perception that they can't go into the pharmacy and just ask a question, or the perception that if they do, it'll be in an unprivate environment. So they* [CHWs] *need to feel that they have like sort of priority access to the pharmacist. They're* [CHWs] *not just another patient, they're a healthcare team member that's helping that patient*.” P31 *14.2. “And knowing which patients are under care of the health navigator and which aren't, because you have no idea of knowing if someone's receiving help or not. Who looks after what clients.”* P24	14.3. “*If pharmacists, (…) care workers or GP work together, and know each other, that would really support… But first of all need to know each other, need to know this community health navigator, it exists.”* P03 *14.4. “(…) that is how the system works, GP then the Allied Health team, then the community navigators, the community workers or health workers, and then the community members. (…) And the BCN* [bilingual care navigator], *they know that so it's always good to work with the Allied Health team members. (…) It is good for the GPs as well to follow‐up because BCN they are connected with the GPs as well as pharmacist. So that will be in complete loop, the BCN will be connected to GP so the GP knows that the person is taking medication from the pharmacist. And so I think the communication comes easier.”* P04 14.5. “*I'm running this program from nearly four to five years. And yeah, people keep coming. (…) So it depends on the grants and funding and everything. So I just liase with my manager in health promotion team, and like and whatever she says, we just deliver program accordingly*.” P04 14.6. “*XXX's being very good at securing, funding is important for the service over a long period of time, which I guess we take for granted. But yeah, (…) People start as a pilot and it goes well, and everyone assumes it's going to go on, but… No, no, it was just the pilot*.” P27

*Note:* The quotes are all verbatim quotes and no grammatical corrections have been done.

#### 3.2.1 Theme 1: A Range of Collaborative Activities

Most pharmacists and CHWs reported a range of collaborative activities from parallel collaboration (i.e., defined when in the absence of CHWs or pharmacists, the pharmacists or CHWs supported CHWs or pharmacy services, respectively) to an interactive collaboration (i.e., defined as a direct contact between CHWs and pharmacists to support the patient).

The collaboration appeared to depend on a multitude of factors, including the local context (e.g., Australia vs. New Zealand, cities and small towns, hospital and community pharmacies). The frequency of the collaboration reported ranged from no collaboration to monthly, weekly or daily collaboration.

Two pharmacists and five CHWs reported that they did not have direct interprofessional collaborations with CHWs and pharmacists, respectively (Quotes 1.1 and 1.2, Table 2). They reported their perceived barriers to the collaboration and strategies to foster the collaborative practices but were not able to reflect on any experiences of collaboration. However, we believe that their perspectives enriched the data and provided further examples of potential challenges and strategies to the CHW‐pharmacist collaboration. While reporting the results, we have distinguished between reported barriers and strategies that were experienced versus those that were not based on experience.

I haven't [worked with CHWs], (…) which is unfortunate, because, (…) I know they exist, but they're not really seen. And you're not really taught, either in university or even out, as a healthcare worker, how you can engage with them.

P32, pharmacist

A few pharmacists mentioned that they only had a few interactions with CHWs (e.g., only communicated during phone conversations to gather some information to make sure that the handover process was clear). Most CHWs in New Zealand reported a daily collaboration with pharmacists, while in Australia, the most frequent reported collaboration was once per week.

We know quite a bit about pharmacy, because we're in and out of there on a daily basis.

P28, CHW

##### 3.2.1.1 CHWs Connect Patients and Pharmacists

Most CHWs and pharmacists reported that CHWs facilitated the link and communication between pharmacists and patients, and that patients felt more comfortable explaining and asking questions when a CHWs was present (Quotes 2.1 and 2.2., Table 2).

It's also quite handy to have the Aboriginal Health Worker [the AHW] come in with you, (…) it sort of breaks down any sort of cultural barriers that there might be and I think the patient's kind of feel a little bit better. They're a bit more responsive to anything you might ask. And obviously, if we're talking about medications, it's quite personal. So they're [the AHW] really good at sort of bridging that gap. And I guess sort of making sure the patient is comfortable that you're there.

P07, pharmacist

CHWs bridged the cultural gap and ensured cultural support to the patient, which facilitated understanding and respect towards the patient. CHWs provided language support, when necessary, but one CHW clearly stated that they were not interpreters. These factors were felt to help build and improve the relationship between pharmacists and patients.

##### 3.2.1.2 CHWs and Pharmacists Share Information

CHWs and pharmacists reported sharing information, such as the CHW bringing messages to the pharmacist or asking questions or seeking advice about the patient's symptoms on the patient's behalf, or the pharmacist wanting to clarify a concern about the patient with the CHW or answering the CHW's queries. One hospital pharmacist commented that the information shared with and by the CHW was documented on the patient's electronic medical record. However, a few pharmacists and CHWs reported that information sharing was limited in order to respect the patient's confidentiality, unless the patient gave his/her standing permission (Quote 3.1. and 3.3., Table 2).

Information that was shared was mostly about medications (e.g., medication list and history, side effects, interactions, medicine issues discovered by the CHW at the patient's home), medication management (e.g., medication adherence, management of dose administration aids [DAAs], scripts, medication pick‐ups), patient's medical condition, the patient's needs, lifestyle (e.g., living conditions, patient's lifestyle history, if the patient benefited from paid care services, financial struggles), and hospitalisations (e.g., the CHW informed the pharmacist that a patient was admitted to the hospital or sent the discharge form to the pharmacist) (Quote 3.2., Table 2).

CHWs and pharmacists reported that they communicated face‐to‐face, by phone, text messages or emails. One community pharmacist reported that emails may not be the best way to communicate with CHWs as they were busy working on site with the patients. A few pharmacists reported that CHWs were more reachable than patients over the phone and it was easier to communicate with CHWs than with patients. A few pharmacists and CHWs reported that communication was mostly informal.

Of note, a few CHWs and pharmacists reported that when appropriate, they collaborated together with carers, the patients' family, general practitioners (GPs), doctors, nurses, social services (e.g., to find a shelter for a homeless patient) and addiction treatment services.

##### 3.2.1.3 CHWs Support the Patient's Medication Management

Most participants reported that the collaboration occurred when CHWs came to the pharmacy to pick‐up the patient's medication. A few CHWs and pharmacists reported that CHWs helped to effectively report patient's medication history (Quote 4.2, Table 2).

So I need to be able to work with them [CHWs] to get the information where they [patients] get their normal medicines from, what they're normally on.

P01, pharmacist

My clients, most of them discharged from the hospital. (…) In order to update the medication list, so I usually call the pharmacist (…). If this client have a lot of issues with the medication, I usually trying to talk to the pharmacy.

P03, CHW

In some situations, CHWs gave medicines to patients, participated in monitoring the use of DAAs by the patient, and monitored for side effects. One CHW working in New Zealand reported that they sometimes returned unused medicines from patients to the pharmacy.

Additionally, a few pharmacists and CHWs reported that there was mutual patient referral, where CHWs referred patients to pharmacists to improve their medication management, and pharmacists referred patients in need of CHWs' services. While in Australia, hospital pharmacists referred patients to CHWs and vice‐versa (Quote 4.1, Table 2), in New Zealand, one CHW stated that the GP—and not the pharmacist—made the official referral to CHWs to include the patient in a CHW programme.

##### 3.2.1.4 CHW Role to Increase Patient Uptake of Pharmacy Services

A few CHWs reported that CHWs encouraged patients to access and benefit from pharmacy services, by prompting patients to have a medication review, and encouraging patients to use DAAs or prompting patients to meet the pharmacist (e.g., for medication issues, to return old medicines to the pharmacy, to ask questions about or utilise pharmacy services) (Quotes 5.1. and 5.2., Table 2).

So maybe it might be that we explain to the navigator how our services work, so they're, in turn, able to explain that to clients.

P30, pharmacist

Sometimes I take the clients visit pharmacies together. (…) If it's like, suggest the clients to do the home medication review, that will (…) take longer. (…) I actually make the client aware there is a service [the home medication review] down there.

P03, CHW

Other CHW roles that increased the patient's uptake of pharmacy services included help navigating patients to the pharmacy, filing documents on the patient's behalf, helping patients plan a GP appointment and contacting doctors so that they send the patient's new scripts to the pharmacy.

##### 3.2.1.5 CHWs Reinforce Pharmacists' Recommendations

Following patient consultation with healthcare providers, including pharmacists, CHWs supported patients' understanding of treatment and medication regimen (Quote 6.2., Table 2). A few pharmacists and CHWs reported that CHWs helped with patient follow‐up, including patient education on medications, reminders for medication adherence, and information on pharmacy services (Quote 6.1., Table 2).

(…) If they're [patients] relying on the support worker entirely, I will just tell the support worker what to do, and, they will then relay the message onto the patient, or write it down for them, or help them remember it, set alarm [to take medications], or something like that.

P15, pharmacist

#### 3.2.2 Theme 2: Perception of Each Other's Roles

Most participants reported that there was a lack of clarity of each other's role (Quotes 7.1. and 7.3, Table 2), which is described further in this article as a challenge to the collaboration. However, pharmacists and CHWs were sometimes able to describe each other's roles (Quotes 7.2. and 7.4., Table 2), though not always accurately.

No one really knows what each other does.

P23, CHW

I think there's a real gap because I see a health navigator, I don't really know what they do, and I think that I'm not alone in that.

P22, pharmacist

One CHW working in Australia believed that pharmacists needed to understand that there is a different approach to patient care, that is pharmacists have more of a clinical approach, but CHWs have a holistic view.

Most CHWs reported that pharmacists were busy and that there was a long waiting time for patients at the pharmacy. Four CHWs suggested that pharmacists should educate patients (e.g., on the medication regimens, on DAAs) or should advise patients on the different subsidy schemes that patients can benefit from at the pharmacy.

Most pharmacists and CHWs acknowledged the benefit of each other's roles. One pharmacist in New Zealand said that pharmacists do need to appreciate the unique skills that CHWs have. Pharmacists talked about CHWs as being advocates for patients, keeping people out of hospitals, being a relevant source of patient information, and being “the glue” or the “liaison point” in the healthcare system (Quote 7.2., Table 2).

I feel community health care workers (…), probably like the glue in the healthcare system, where they kind of do all that support work. And it's hard to kind of pinpoint down to one particular activity that they do, because they do such a wonderful array of things, and I'm sure a lot of them go above and beyond what's expected of them.

P18, pharmacist

One pharmacist said that CHWs empowered patients to maintain their autonomy. Pharmacists reported that CHWs cared about patients—they were empathetic, supportive, professional, knowledgeable and organised, and they had interpersonal and life skills to offer. Two pharmacists reported that CHWs knew the patient better than pharmacists and two other pharmacists explained that CHWs knew when something was not right with a patient.

CHWs (among them, one who did not collaborate with pharmacists) talked about pharmacists as being experienced, knowledgeable, experts in medications and that they understood the patients' needs. Pharmacists were welcoming and willing to help and easily available for people (Quote 7.5, Table 2).

#### 3.2.3 Theme 3: Challenges to Collaboration

##### 3.2.3.1 Lack of Clarity Regarding Each Other's Roles

There was a lack of clarity regarding each other's roles and scope of practice, especially by pharmacists towards CHWs' roles and CHW services available to patients (Quote 8.1, Table 2).

Pharmacists used a multitude of umbrella terms to characterise CHWs (e.g., aboriginal health workers, paid carers, CHWs in drug and alcohol, disability carers, health care support workers, health navigators, multicultural community liaison). One community pharmacist working in Australia reported to be unclear about who employs CHWs and another pharmacist also working in Australia reported that CHWs' roles varied according to the employer they worked for. Another pharmacist who did not collaborate with CHWs reported to be unsure about CHWs' academic qualifications.

One CHW in Australia was confused about pharmacists' qualifications and a few CHWs, among them one who did not collaborate with pharmacists, reported a lack of knowledge of the pharmaceutical services available (Quote 8.5., Table 2).

One academic pharmacist reported fearing that CHWs may make some clinical decisions instead of the pharmacist, and that this could impact the pharmacist‐patient rapport. Likewise, a CHW reported experiencing resistance towards their navigation roles by clinicians (e.g., doctors) (Quote 8.6., Table 2).

(…) because I know as a health coach, there was a bit of resistance with the doctors I worked with, but then eventually they kind of understood my role.

P17, CHW

A community pharmacist in New Zealand reported that there was a power imbalance, a hierarchy between pharmacists who were registered healthcare providers, and CHWs, who were not necessarily qualified. More than half of the pharmacists reported that CHWs had low education, poor clinical skills and training (Quote 8.2, Table 2). Two pharmacists reported that CHWs may lack some knowledge of the healthcare system, medicine prices and the process of prescription, medicines dispensing and collection from pharmacies. A pharmacist reported that there is no training on how pharmacists can collaborate with CHWs.

CHWs and pharmacists expected more from each other. A few pharmacists reported that the current involvement of CHWs was inadequate. They believed that the quality of the collaboration depended on the level of involvement of the CHWs and pharmacists and their motivation to collaborate (Quote 8.3 and 8.4, Table 2).

So that's kind of at one end of the spectrum, but there are some [CHWs] that really barely communicate with us at all, and then, then at the other end of the spectrum, it's the opposite, that they're giving us a lot of the information that we would need to be able to adjust our care for the patient.

P14, pharmacist

For instance, pharmacists reported that CHWs had not returned their calls or showed up. One CHW in Australia reported that he/she would expect pharmacists to be more involved in co‐designing educational programmes for patients.

A few pharmacists explained that there was a lack of knowledge on the benefits of working with CHWs, and that the CHW‐pharmacist collaboration was not common or well known. One pharmacist explained that collaboration would be difficult if pharmacists or doctors lacked respect for CHWs, or they felt that CHWs were inexperienced. A few pharmacists reported that CHWs were not included in healthcare teams and were not represented in meetings with pharmacists and healthcare providers. One pharmacist felt that not collaborating with a CHW was a missed opportunity as they have useful patient information to share with pharmacists.

##### 3.2.3.2 Poor Contact and Communication

One of the barriers to collaboration reported was the lack of contact between CHWs and pharmacists (e.g., they did not have each other's direct phone contact, CHWs did not know which pharmacist was available to report to). A few pharmacists reported that there were only limited communication channels (mostly via phone), and one pharmacist reported that CHWs sometimes used a particular computer programme to edge their notes into, that the hospital pharmacist cannot access, which prevents efficient communication.

Additionally, CHWs and pharmacists reported that a working relationship was not established. One pharmacist who did not collaborate with CHWs said that CHWs did not usually make themselves known. Conversely pharmacists did not normally reach out to CHWs (Quote 9.1, Table 2). For instance, a pharmacist reported that sometimes they did not know if a CHW was the patient's CHW or the patient's relative, and a few pharmacists reported that they did not know if a CHW was involved in the patient's care.

Some of the barriers are that we don't see some of them [CHWs]. So, I assume quite a few of our patients do have carers or health workers, but we actually don't see them. So I dare say, a lot of our patients have support, but I don't know who they are or even how to contact them if they do.

P16, pharmacist

One CHW working in New Zealand explained that they popped into the pharmacy to pick up the patient's scripts but without further communication or collaboration with pharmacists.

Experiences of miscommunication were reported by both pharmacists and CHWs (Quote 9.2, Table 2).

Almost half of the pharmacists reported that patients may have multiple CHWs and carers, which can add confusion and risk for pharmacists, and may weaken their relationship with CHWs. Indeed, one pharmacist reported that CHWs can be casual contingency workers and may not be permanently in charge of the same patient.

Besides, CHWs got burnt out quickly, leading to high staff turn‐over. Of note, one CHW in New Zealand reported that pharmacy staff turn‐over was also a barrier because new pharmacy staff didn't know CHWs and their services, which sometimes led to CHWs not being able to pick‐up medications on patients' behalf.

##### 3.2.3.3 Organisational Barriers

There were a number of organisational barriers to collaboration reported. These included unavailability of the pharmacist and consequent CHW communication with pharmacy retail staff not involved in patient care; long waiting times at the pharmacy; short‐staffed pharmacies limiting pharmacist times with CHWs; and community pharmacies not being suitable environments for collaboration (Quote 10.1, Table 2).

But other than how busy they [pharmacists] are, and it's like, so chaotic when you're in there and you don't want to feel like you're wasting their time because everyone's moving around. Yeah, I don't. Maybe that would be a barrier for some people, not wanting to be an annoyance or feel like they're an annoyance, maybe.

P26, CHW

Another barrier occurred when the prescriber did not collaborate with pharmacists and CHWs.

A few pharmacists and CHWs explained that there were obstacles to the collaboration when the patient was not happy to be supported by a CHW (Quote 10.2., Table 2), the patient misunderstood the pharmacist's roles, the patient did not want to see the pharmacist, or that the CHW‐patient relationship was not based on trust.

Pacific patients don't know that they can go and ask the pharmacist to help them like they don't understand that. They think the doctor has said this, I'm just gonna take it. (…) So they [Pacific patients] just believe that the pharmacy people, pharmacists just release them the medication.

P17, CHW

##### 3.2.3.4 Lack of Collaborative Practice Guidelines

The referral process between CHWs and pharmacists was not documented (Quote 11.1, Table 2). One CHW working in Australia said that he/she did not get paid for linking patients to pharmacists (Quote 11.2, Table 2).

(…) but it's not sort of documented anywhere, it's just us calling on the phone.

P07, pharmacist

Normally I just refer to pharmacies because (…) I don't get paid for orders, liaising and all those things, you know, so I don't get involved unless until, like, you know, if they really want somebody from language specific or something, and if I know some pharmacists, then maybe I can just link that with that person.

P04, CHW

#### 3.2.4 Theme 4: Strategies to Collaboration

##### 3.2.4.1 Clarification of Roles

To improve the collaborative practice, a few pharmacists and CHWs suggested clarifying each other's roles, scope of practice and the expectations of each other, especially CHWs' roles and services (Quotes 12.1. and 12.4., Table 2).

I think pharmacists do need to appreciate the unique skills that navigators have and understand almost a case of walking in their shoes to understand what it's like to do their job successfully.

P30, pharmacist

One pharmacist suggested representation of CHWs, where they advocated for CHWs, so that clinicians would be able to better understand what CHWs are capable of. Some pharmacists suggested revising the curriculum of pharmacy degree programmes to promote the CHW‐pharmacist collaboration—and CHWs' roles—through education and training. For instance, case studies and workshops could be delivered by pharmacists and CHWs and interprofessional shadowing can be implemented as part of interprofessional learning (Quotes 12.2. and 12.5., Table 2).

I think (…) interprofessional learnings would be good. So actually having case scenarios with either doctors, nurses and community health workers and getting their role, (…) having actual practice in community healthcare workers coming in to the classroom and explaining their role and what they do, and again, maybe even having like workshops on it, about when you would seek out a worker or not, or you can kind of solve a problem by yourself.

P32, pharmacist

Both of them need to (…) learn more about this role. Educate them about role, because it is so important role. But if the pharmacist don't know, they will be not interested.

P09, CHW

Indeed, a pharmacist suggested shadowing each other, which will allow spending time together to better understand and appreciate each other's roles on the ground and ultimately learning from each other's roles experientially rather than theoretically. A pharmacist suggested standardising CHW training on the same level as a pharmacy technician.

Some pharmacists suggested that raising awareness of the benefits of CHW‐pharmacist collaboration along with positive testimonials would foster the collaborative practice. Participants felt that there could be more dissemination and information provided about CHW‐pharmacist collaboration and CHW roles through conferences, media, social media, to GPs, carers and volunteers.

One community pharmacist in Australia explained that it would be important to have a standardised CHW‐pharmacist collaborative practice. One hospital pharmacist even recommended having a mandatory referral process between CHWs and pharmacists (Quote 12.3., Table 2).

##### 3.2.4.2 Improving the Working Relationship

Pharmacists and CHWs recommended getting to know each other, introducing themselves, particularly to new staff, making themselves available to help each other, and supporting each other to build and maintain the professional relationship with trust (Quote 13.2., Table 2).

We [CHWs, are] kind of sometimes their [pharmacists'] eyes and ears out there.

P28, CHW

So I would say to the new navigator, organize a time to meet the pharmacist, let them know what you do, how to contact you, establish that relationship first, so that you can deal with them on a one on one basis.

P22, CHW

CHWs from New Zealand reported that having a good working relationship with pharmacists, mostly face‐to‐face, has helped them to collaborate. Most CHWs and pharmacists in New Zealand reported that building this CHW‐pharmacist relationship might be easier in small towns as people knew each other better than in larger cities.

Three pharmacists reported that it would help the collaboration to adopt a ready to change attitude. Importantly, one CHW highlighted that collaboration should involve an open discussion between two professionals to avoid power imbalance. Both CHWs and pharmacists suggested fostering more interactions between pharmacists and CHWs by gathering in same place of work—CHWs in pharmacy or pharmacists reach out to the organization that employs CHWs. Three pharmacists reported that they should engage with a CHW early when a patient needed it (e.g., when discussing medication management plans with the patient).

A few pharmacists and CHWs suggested organising interdisciplinary team meetings to update patient information through videoconferencing or face‐to‐face meetings. A few pharmacists suggested that CHWs should be considered as part of the healthcare team.

Pharmacists recommended implementing a shared online communication platform, such as My Health Record in Australia, that the healthcare team could access. Most pharmacists suggested having a shared list of CHWs' and pharmacists' contact details to facilitate communication (Quote 13.1., Table 2). A pharmacist also suggested creating a newsletter to make CHWs' contacts available to pharmacists and a leaflet about CHW services to the pharmacists.

##### 3.2.4.3 Organisational Facilitators

At the pharmacy level, one community pharmacist suggested that CHWs should be given priority access to pharmacists (Quote 14.1., Table 2).

They [CHWs] probably know the patient better than us (…). So they [CHWs] need to feel that they have like sort of priority access to the pharmacist. They're [CHWs] not just another patient, they're a healthcare team member that's helping that patient.

P31, pharmacist

Furthermore, a few pharmacists reported that they should know when a CHW is involved in a patient's care (Quote 14.2., Table 2). In parallel, one pharmacist reported that CHWs should clearly identify themselves at the pharmacy. One hospital pharmacist suggested notifying CHW involvement in health summaries with GPs, and two pharmacists suggested asking patients if a CHW was involved in their care.

A few pharmacists suggested that pharmacists can provide education to CHWs and upskill them to improve their health and medication knowledge (e.g., on common health conditions, non‐medical ways to support patients, how medications work), which will facilitate collaborative practice.

One pharmacist in Australia explained that having a pharmacist “champion” within the hospital setting advocating for CHW‐pharmacist collaboration has been a facilitator. One CHW in New Zealand reported that a facilitator was to engage with a key pharmacist as the person of reference at the community pharmacy.

One CHW in Australia reported that collaborative practice was facilitated when patient and pharmacist already knew each other. Interprofessional collaborations with hospital and community pharmacists, carers, CHWs, healthcare workers, doctors and patients were recommended (Quotes 14.3 and 14.4, Table 2). To facilitate this, two pharmacists explained that having a chronic care coordinator was successful in connecting health care professionals and CHWs for patient care.

Lastly, a few pharmacists suggested recruiting more CHWs and allowing extra human resources (CHWs and pharmacists) to allow more time for effective collaboration. One pharmacist reported that there should be a budget allocation for implementing programmes that would allow a collaborative practice between CHWs and pharmacists.

A CHW working in Australia explained that CHWs' job depends on funding; whereas one CHW working in New Zealand explained that funding for their CHW programme has been secured over time (Quotes 14.5 and 14.6, Table 2).

## 4 Discussion

### 4.1 Summary of the Findings

This is the first qualitative study reporting on CHW‐pharmacist collaborative practices in Australia and New Zealand. The findings suggest that collaboration does exist, but the extent varied, and ranged from a CHW linking the patient to the pharmacist to CHWs supporting uptake of pharmacy services. This study also identified that a better clarity regarding each other's roles influences the collaboration and that CHWs and pharmacists acknowledged the benefit of each other's roles, but reported that there is a need to improve their knowledge of the mutual benefits of collaboration, and to standardise CHW‐pharmacist collaborative practice.

### 4.2 CHW Roles in Optimisation of Pharmaceutical Services

Both interactive (primarily occurring at the community pharmacy) and parallel collaboration (mainly where the CHWs were at the patient's home) ensured the continuity of patient care. Participants believed that CHW‐pharmacist collaboration facilitated improved access to medicines by disadvantaged patients, better understanding of patient needs and optimised pharmaceutical services delivered by pharmacist. Indeed, this study highlighted that, in the presence of CHWs, patients may feel more comfortable and safer to share their experiences, questions and doubts about their disease and medication management, and this, in turn, can facilitate patients' willingness to collaborate with pharmacists. Ultimately, CHW‐pharmacist collaboration may increase the number of patients who benefit from pharmaceutical services.

However, the implementation of CHWs' roles within pharmacies is a relatively new concept. A recent study demonstrated the feasibility of a health‐related social needs screening in a community pharmacy where patients were referred to an embedded CHW [40]. The CHW‐pharmacist collaboration successfully addressed health‐related social needs [40]. CHWs and pharmacists working in the same environment can foster the CHW‐pharmacist collaborative practice, as also mentioned by participants in our study.

### 4.3 Perceptions, Challenges and Strategies

The perceptions of collaboration varied among participants. For instance, while simply picking up a DAA at the pharmacy was not considered as a collaboration, some other did consider this to be a type of collaboration.

Parallel and interactive collaboration can be supported by standardised practice and the clarity regarding each other's roles and expectations. As a first step, pharmacists' and CHWs' umbrella associations, if the latter do exist, should meet and discuss how to foster and implement CHW‐pharmacist collaboration, and establish guidelines for such collaboration. The standardisation of the CHW and pharmacist's roles within the collaborative practice may improve communication, bring clarity on the information sharing process, improve interprofessional relationship and decrease a power imbalance between them.

Notably, CHWs and pharmacists acknowledged the benefit of each other's roles. In the literature, pharmacists have been shown to have advocated for CHWs' roles, stating that they can improve patient‐pharmacist communication, medication adherence and assessment of patient health condition [25]. In another study, almost 90% of pharmacists with experience with a CHW agreed that adding a CHW to the healthcare team was beneficial to patients [26]. Another study showed that CHWs felt empowered, had increased self‐efficacy and confidence when they worked with pharmacists in an interprofessional medication therapy management programme for chronic disease [27]. Indeed, role clarification is fundamental to good collaborative practice [3] and should be promoted as part of the training of CHWs and pharmacists.

Some of the barriers identified in our study were previously reported, such as the power imbalance between CHWs and pharmacist [25], poor communication between them [25, 26, 27] and the confusion of pharmacists regarding CHWs' roles [25]. The lack of clarity around each other's role may also prevent their collaboration and is highlighted by our findings that CHWs and pharmacists expected more from each other, or pharmacists feared that CHWs would encroach on their roles.

The lack of a standardised practice for CHW‐pharmacist collaboration may explain the lack of collaboration or consistent collaboration. Whereas the collaboration between CHWs and health professionals like physicians and nurses has been reported previously [9], CHWs and pharmacists lack guidance on how to contact each other, how to meet, what types of information to share, and what types of services to deliver jointly for patients. In a previous study, pharmacists were asked if they have a standardised method for sharing information with CHW, and only 62% agreed [26], illustrating a lack of standardisation for collaboration.

The patient should be at the centre of collaborative practice between CHWs and pharmacists. This may act either as a barrier (e.g., when the patient does not want to be supported by a CHW or a pharmacist) or facilitator (e.g., when the patient already has a trusted relationship with pharmacist or CHW). Promoting pharmacists' and CHWs' roles would improve recognition of their roles by patients, which might also result in patient benefits from pharmaceutical and CHWs services. Further research is needed to explore (i) existing CHWs' roles and effectiveness of their interventions, (ii) CHW‐pharmacist collaborative experiences in local contexts in order to know more on how CHWs can help pharmacists and vice‐versa, (iii) patient's perceptions on CHW‐pharmacist collaboration.

No participant was able to suggest the most effective way for CHWs and pharmacists to work together. They identified barriers and suggested some ways that these barriers should be addressed. While some barriers can be addressed locally using some implementation strategies in the short term (e.g., a frequent catch‐up meeting), some other barriers would require policy changes (e.g., increasing funding for CHW services and institutional support [41]).

There is a need to co‐design CHW‐pharmacist interventions involving local patient and public representatives to study and build evidence on the impact of CHW‐pharmacist collaboration on pharmacy service use and patient health outcomes; and ultimately, co‐designing the collaborative practice model of care to standardise the collaboration.

### 4.4 Study Limitations

This qualitative study has some limitations. Firstly, the results are not expected to be generalised beyond the group of participants interviewed. Our sample is not a random sample of the population but was mostly restricted to the researchers' and health professionals' networks through the snowballing recruitment approach.

Secondly, there is the potential for social desirability bias—where participants may have reported ideas that they would think were socially acceptable or expected by the researcher [42].

## 5 Conclusions

There is a range of CHW‐pharmacist collaborative activities reported by participants in Australia and New Zealand. Collaboration occurred when the CHW connected the patient to the pharmacist, for instance by bridging the cultural gap or facilitating the communication. As a result, relevant information was shared, and uptake of pharmacy services was enhanced by the CHWs.

The lack of clarity regarding each other's roles, poor contact and communication between CHWs and pharmacists and the lack of standardised collaborative practice guidelines were challenges to the collaboration. CHWs' and pharmacists' roles need to be clarified among healthcare providers and patients. Raising awareness about the mutual benefits of collaboration through training and education and standardising the roles within the CHW‐pharmacist collaborative practice were recommended.

Fostering the CHW‐pharmacist collaboration may lead to improved patient access to pharmaceutical services, addressing their social determinants of health and ultimately improving their health outcomes.

## Author Contributions

**Carole Bandiera:** conceptualisation, investigation, funding acquisition, writing – original draft, methodology, visualisation, writing – review and editing, formal analysis, project administration. **Megan Darwood:** investigation, writing ‐ review and editing, formal analysis. **Sabuj Kanti Mistry**, **Elizabeth Harris**, **Mark F Harris:** conceptualisation, investigation, methodology, validation, visualisation, writing – review and editing, formal analysis. **Parisa Aslani:** conceptualisation, investigation, methodology, validation, visualisation, writing – review and editing, formal analysis, project administration, data curation, supervision.

## Ethics Statement

The study was conducted in accordance with the declaration of Helsinki and was approved by the Human Research Ethics Committee of the University of Sydney (2024/HE000334).

## Consent

Written and informed consent was obtained before the start of data collection.

## Conflicts of Interest

The authors declare no conflicts of interest.

## Supporting information

SM1 COREQ checklist.

SM2 Interview guide for CHWs.

SM3 Interview guide for pharmacists.

SM4 Demographic data form.

## Data Availability

The datasets generated and analysed during the current study are not publicly available due to the approval obtained from the Human Research Ethics Committee of the University of Sydney.
