# Supplementary material for: Interprofessional Collaboration Between Community Health Workers and Pharmacists
Source: Health Expect. 2026 Jan 19;29(1):e70538. doi: 10.1111/hex.70538 (PMC12815246; doi:10.1111/hex.70538)
Supplement: Supplementary file 3 — SM3 Interview guide for pharmacists. [file HEX-29-e70538-s002.pdf]

Semi-structured Interview Guide for pharmacists

Aim	Questions	Field Notes
**Welcoming the pharmacist**	*Hello Ms/Mr, as written in the participant information sheet, we are going to discuss about your interprofessional collaboration work with community health workers. The duration of this interview will approximatively be 30 minutes.*<br>*I would like to remind you that the data collected are coded (i.e., de-identified) and the audio-recording will be destroyed after data transcription.*<br>*There is no right or wrong answer, this interview will help us to better understand how you work with community health workers and what are the barriers and facilitators you have encountered to working with community health workers in your daily practice.*	
**Professional Background**	*You are a community/hospital pharmacist.*<br>▪ Could you please explain to me your professional journey as a pharmacist (i.e., community or hospital pharmacist, years of practice and setting)?<br>▪ How many years have you worked as a pharmacist?<br>▪ What are your main roles and responsibilities in your current position?<br><br>*I would like to focus on a specific group of patients or customers that come to your pharmacy. These are underserved or vulnerable patients.*<br>▪ Thinking about this group of people, what are barriers you have encountered when delivering pharmaceutical services?<br>▪ Thinking about this group of people, what facilitates or helps you deliver pharmaceutical services?	
**Perceived Roles of community health workers**	As part of your daily practice as a pharmacist, you may work with community health workers.<br>▪ What do you know about community health workers and their roles and responsibilities?<br>▪ Could you describe for me the types of services that community health workers deliver in their daily practice?<br>▪ What do you believe are the most important tasks community health workers do?	
**Interprofessional collaborations with**	*You probably work in several interdisciplinary health teams, such as with general practitioners, nurses, social workers. In this study, we would like to understand how you work and collaborate with community health workers.*	

**community health workers**	▪ How would you describe your typical interactions or communications with community health workers in your daily work (formal and informal interactions)?<br>▪ How often do you collaborate together?<br>▪ What types of services do you deliver together?<br>▪ What type of patient information do you mutually share?<br>▪ How do you think working with community health workers has impacted patient health outcomes?<br>▪ Can you provide one example of a collaboration with a community health worker that positively impacted patients' health outcomes?<br>▪ Can you provide one example of an unsuccessful collaboration that negatively impacted patients' health outcomes?	
**Proposition of a model of care for a collaborative practice from the scientific literature**	*A recent study from the USA suggested a model of care for a collaborative practice between community health workers and pharmacists. [The investigator CB shows the model below to the pharmacist, and explains it out loud]*<br>• What do you think about it?<br>• How do you think this model would work in Australia [*respectively New Zealand*]?	

<table>
<tr>
<td></td>
<td>

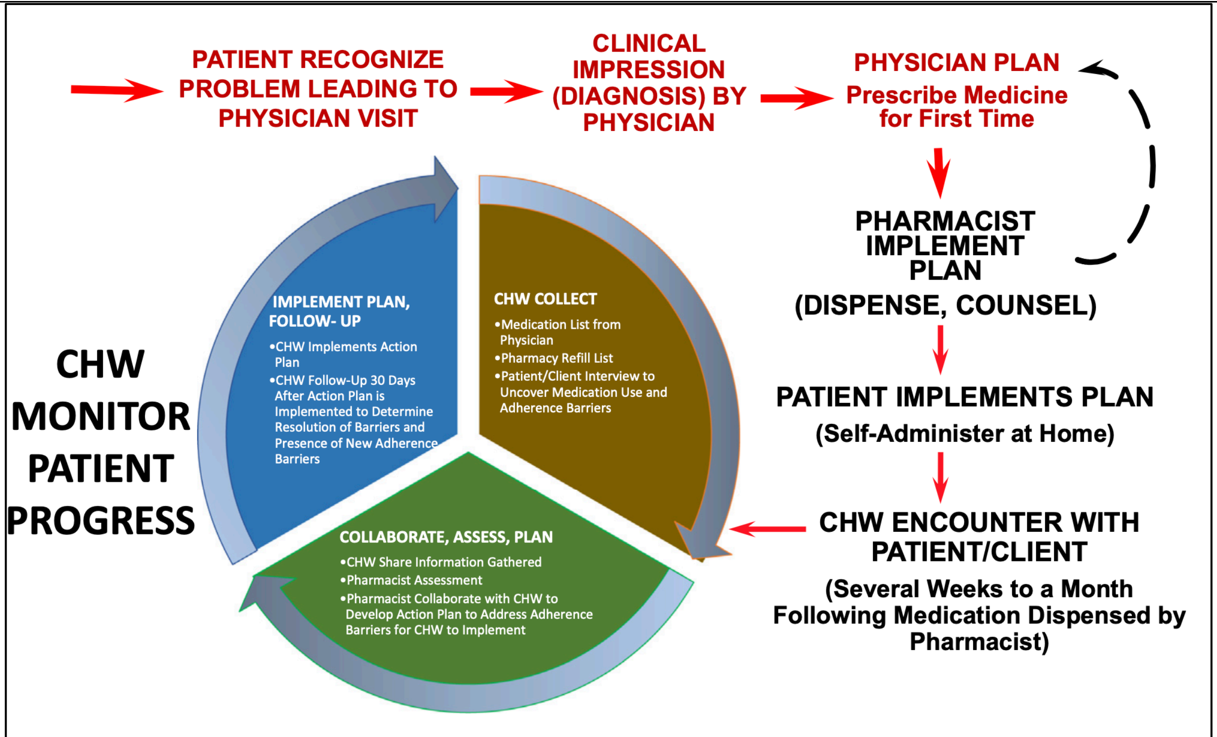

Figure 1. Collaborative CHW-pharmacist practice model. Abbreviation used: CHW, community health worker.

Segal et al., Opportunities and responsibilities for pharmacists to improve their effectiveness in addressing medication adherence through culturally sensitive collaborations with community health workers, J Am Pharm Assoc, 2020, DOI: 10.1016/j.japh.2020.02.023

</td>
<td></td>
</tr>
<tr>
<td>

Barriers to the interprofessional collaboration

</td>
<td>

We will now explore the barriers to this interprofessional collaboration.

- What barriers have you experienced, and what do you think would be barriers that prevent an effective collaboration with community health workers (systemic, organisational or other)?
- Have you encountered any difficulties in communicating with community health workers?
- Have you encountered any difficulties in sharing information with community health workers?

</td>
<td></td>
</tr>
</table>

	▪ How do you think these barriers could be addressed?<br>▪ What strategies do you use to overcome these barriers in your daily practice?	
**Facilitators of the interprofessional collaboration**	*We will now explore the facilitators of this interprofessional collaboration.*<br>▪ What facilitators have you used, and what do you think you could use as facilitators of the interprofessional collaboration?<br>▪ How do you think these facilitators could be strengthened?<br>▪ How would you think that the facilitators can address the barriers cited previously?<br>▪ What support or resources would facilitate the collaboration with community health workers?	
**Perspectives in practice for the future**	*We are trying to understand how pharmacists and community health workers can work synergistically together.*<br>▪ If you were to meet another pharmacist, what would be your recommendations to improve the interprofessional collaboration between that pharmacist and community health workers?<br>▪ If you imagine yourself working in the next 10 years with community health workers, what changes in your daily practice would you implement to improve the collaboration?	
**End of the interview**	*We have xx minutes left.*<br>▪ Is there anything else we have not talked about that you would like to discuss now?<br>▪ Do you have any further comments?	
