# Supplementary material for: Interprofessional Collaboration Between Community Health Workers and Pharmacists
Source: Health Expect. 2026 Jan 19;29(1):e70538. doi: 10.1111/hex.70538 (PMC12815246; doi:10.1111/hex.70538)
Supplement: Supplementary file 4 — SM4 Demographic data form. [file HEX-29-e70538-s001.pdf]

Demographic Data of the Participant

Research Study: EXPLORING THE INTERPROFESSIONAL COLLABORATION BETWEEN PHARMACISTS AND COMMUNITY HEALTH WORKERS IN AUSTRALIA AND NEW ZEALAND: THE INTER-PHARM-CHWs QUALITATIVE STUDY

Participant Code ___________

Which of the following best describes your gender?
☐ Man ☐ Woman ☐ Trans and/or gender diverse ☐ I use a different term (this is inclusive of culturally-relevant terms for the expression and/or identification of your gender, if applicable) ☐ Prefer not to say

Year of birth: _________

Months/Years of work experience in health: _______ months / ______ years

Cultural Background[1]:

☐ OCEANIAN
☐ NORTH-WEST EUROPEAN
☐ SOUTHERN AND EASTERN EUROPEAN
☐ NORTH AFRICAN AND MIDDLE EASTERN
☐ SOUTH-EAST ASIAN
☐ NORTH-EAST ASIAN
☐ SOUTHERN AND CENTRAL ASIAN
☐ PEOPLES OF THE AMERICAS
☐ SUB-SAHARAN AFRICAN
☐ Other, as defined by the participant:_____________________________________
☐ Prefer not to tell

Date of the interview ___/___/___

1. Australian-Bureau-of-Statistics. *Australian Standard Classification of Cultural and Ethnic Groups (ASCCEG)*. 2019 [cited 2024 27.03.2024]; Available from: https://www.abs.gov.au/statistics/classifications/australian-standard-classification-cultural-and-ethnic-groups-ascceg/latest-release.
